# Prediction of Multiphase Flow in Ruhrstahl–Heraeus (RH) Reactor

**DOI:** 10.3390/ma18133149

**Published:** 2025-07-02

**Authors:** Han Zhang, Hong Lei, Yuanxin Jiang, Yili Sun, Shuai Zeng, Shifu Chen

**Affiliations:** 1 Key Laboratory of Electromagnetic Processing of Materials, Ministry of Education, Northeastern University, Shenyang 110819, China; 2010575@stu.neu.edu.cn (H.Z.);; 2 School of Metallurgy, Northeastern University, Shenyang 110819, China; 3 School of Chemistry and Chemical Engineering, Suzhou University, Suzhou 234000, China

**Keywords:** RH, multiphase flow, gas domain, Euler–Euler model, OpenFOAM

## Abstract

Splashed droplets in the vacuum chamber play an important role in decarburization and degassing in Ruhrstahl–Heraeus (RH), but the scholars do not pay attention to the behaviors of splashed droplets. Thus, it is necessary to propose a new method to investigate the splashed droplets. A Euler–Euler model and the inter-phase momentum transfer are applied to investigate the interaction between the molten steel and the bubbles, and the gas domain in the vacuum chamber is included in the computational domain in order to describe the movement of the splashed droplets. Numerical results show that the flow field predicted by Euler–Euler model agrees well with the experimental data. There is a higher gas volume fraction near the up-snorkel wall, the “fountain” formed by the upward flow from the up-snorkel exceeds 0.1 m above the free surface, and the center of the vortex between the upward stream and the downward stream is closer to the upward stream in the vacuum chamber.

## 1. Introduction

Ruhrstahl–Heraeus (RH) plays an important role in steelmaking processes because it has diversified functions: dehydrogenation, decarburization, denitrification, alloying, inclusion removal, and temperature control [1,2,3]. In RH, the vacuum can lift molten steel in the ladle to the vacuum chamber via immersed snorkels, and the upward flow of the molten steel is driven by argon bubbles [4,5,6]. When the bubbles escape from the free surface of the molten steel in the vacuum chamber, there are lots of molten-steel droplets above the free surface because of the breakage of the bubbles. It should be emphasized that the movement behavior of the droplets play important roles in decarburizing and degassing. In order to have a deep insight into such a complex metallurgical process, a water model and a numerical simulation have been extensively employed to investigate the spatial distribution of bubbles [7,8,9,10,11], the mixing time [12,13], and the decarburization behavior [14,15,16].

The research about the fluid flow in RH can be divided into four types:

(1) In 1989, Tsujino et al. [17] investigated the flow field of molten steel in RH on the base of the flux balance between the down and up snorkel, but the fluid only involves in the molten steel.
(2) The full-buoyancy model was utilized to predict the fluid flow in RH based on the known spatial distribution of bubbles [15,18,19].
(3) The molten steel and the bubbles were continuous phases, and the Euler–Euler model was applied to describe the multiphase flow in RH [20].
(4) The fluid of volume method (VOF) was applied to describe the molten-steel flow, and the discrete-phase model (DPM) was applied to describe the bubbles’ motion in RH [21,22,23,24,25,26,27].

The above studies show the great progress that has been made in studying the flow field in RH, but the full-buoyancy model and the Euler–Euler model treated the free surface of the molten steel in the vacuum chamber as a flat surface, so related papers did not discuss the behaviors of splashed drops in the vacuum chamber. The VOF could trace the movement of the free surface but could not trace the splashed drops in the vacuum chamber because the fact that there are too many splashed drops leads to a heavy computational load. It should be noted that the behaviors of splashed drops are very important for the decarburization in RH. Thus, in the current paper, the Euler–Euler model and an inter-phase momentum transfer are introduced to describe the interaction between the molten-steel phase and the gas phase, and the gas domain in the vacuum chamber is added into the computational domain. In this way, we can have a deep insight into the behaviors of splashed drops in the vacuum chamber. This work is organized as follows: Section 2 gives the grid system and the mathematical model, Section 3 validates the numerical results and determines the mesh number, and Section 4 gives the spatial distribution of the fluid velocity and the gas volume fraction.

## 2. Mathematical Model

### 2.1. Assumptions

The Euler–Euler method is introduced to describe the flow of the molten-steel phase and gas phase in RH. In order to simplify the mathematical model about the multiphase transfer phenomena, the following assumptions are made:

(1) The molten steel and the argon gas are incompressible Newtonian fluids [28,29,30,31,32,33].
(2) The pressure in the vacuum chamber and the fluid temperature are the constants during the RH vacuum-refining process [1,7,9,11,13].
(3) The fluid flow ignores the impact of a top slag and chemical reactions [7,9,18,28].
(4) The bubbles are the rigid spheres [28], and they do not aggregate with each other or break up.

### 2.2. Governing Equations

#### 2.2.1. Euler–Euler Model

The Euler–Euler model consists of the continuity and momentum equation of the gas phase and molten-steel phase [9,29,34].

(1) $$\nabla \cdot (\alpha_{g}\rho_{g}{\vec{u}}_{g})=0$$

(2) $$\nabla \cdot (\alpha_{l}\rho_{l}{\vec{u}}_{l})=0$$

(3) $$\nabla \cdot \;(\alpha_{g}\rho_{g}{\vec{u}}_{g}{\vec{u}}_{g})=-\alpha_{g}\nabla p+\nabla \cdot \left[\alpha_{g}\mu_{\mathrm{eff},g}(\nabla {\vec{u}}_{g}+{\left(\nabla {\vec{u}}_{g}\right)}^{T})\right]+\alpha_{g}\rho_{g}\vec{g}+{\vec{R}}_{g}$$

(4) $$\nabla \cdot \;(\alpha_{l}\rho_{l}{\vec{u}}_{l}{\vec{u}}_{l})=-\alpha_{l}\nabla p+\nabla \cdot \left[\alpha_{l}\mu_{\mathrm{eff},l}(\nabla {\vec{u}}_{l}+{\left(\nabla {\vec{u}}_{l}\right)}^{T})\right]+\alpha_{l}\rho_{l}\vec{g}+{\vec{R}}_{l}$$

where $\alpha_{g}$ and $\alpha_{l}$ are the gas and liquid volume fraction.

(5) $$\alpha_{g}+\alpha_{l}=1$$

The subscripts g and l are gas and liquid, respectively. ρ and $\vec{u}$ are the density and velocity, respectively, kg/m^3^, m/s. $\mu_{\mathrm{eff}}$ is the effective viscosity, Pa·s. $\vec{g}$ is the gravity acceleration, m/s^2^. The pressure *p* is shared by the gas phase and the molten-steel phase, Pa. $\alpha_{g}$ and $\alpha_{l}$ are the effect of the pressure field on the gas phase and the molten-steel phase, respectively. $\vec{R}$ is the inter-phase momentum transfer source term, N/m^3^.

#### 2.2.2. Inter-Phase Momentum Transfer

For the multiphase flow in RH, there is a momentum transfer between the molten steel and the gas [33,34]. The inter-phase momentum transfer source term consists of the drag force ${\vec{F}}_{D}$, N/m^3^; the lift force ${\vec{F}}_{L}$, N/m^3^; the virtual mass force ${\vec{F}}_{\mathrm{VM}}$, N/m^3^; the turbulence dispersion force ${\vec{F}}_{\mathrm{TD}}$, N/m^3^; and the wall lubrication force ${\vec{F}}_{\mathrm{WL}}$, N/m^3^.

(6) $${\vec{R}}_{l}=-{\vec{R}}_{g}={\vec{F}}_{D}+{\vec{F}}_{L}+{\vec{F}}_{\mathrm{VM}}+{\vec{F}}_{\mathrm{TD}}+{\vec{F}}_{\mathrm{WL}}$$

(7) $${\vec{F}}_{D}=\frac{3}{4}\alpha_{g}\rho_{l}\frac{C_{D}}{d_{g}}\left|{\vec{u}}_{g}-{\vec{u}}_{l}\right|\left({\vec{u}}_{g}-{\vec{u}}_{l}\right)$$

(8) $${\vec{F}}_{L}=-\alpha_{g}\rho_{l}C_{L}({\vec{u}}_{g}-{\vec{u}}_{l})\times (\nabla \times {\vec{u}}_{l})$$

(9) $${\vec{F}}_{\mathrm{VM}}=\alpha_{g}\rho_{l}C_{\mathrm{VM}}(\frac{d{\vec{u}}_{g}}{dt}-\frac{d{\vec{u}}_{l}}{dt})$$

(10) $${\vec{F}}_{\mathrm{TD}}=-\frac{3}{4}C_{D}\left|{\vec{u}}_{g}-{\vec{u}}_{l}\right|\frac{\mu_{t,l}}{0.9d_{g}}\alpha_{g}(\frac{\nabla \alpha_{g}}{\alpha_{g}}-\frac{\nabla \alpha_{l}}{\alpha_{l}})$$

(11) $${\vec{F}}_{\mathrm{WL}}=-\alpha_{g}\rho_{l}C_{\mathrm{WL}}{\left|({\vec{u}}_{g}-{\vec{u}}_{l})-\left[({\vec{u}}_{g}-{\vec{u}}_{l})\cdot {\vec{n}}_{W}\right]{\vec{n}}_{W}\right|}^{2}{\vec{n}}_{W}$$

where $d_{g}$ is the bubble diameter, m. $\mu_{t,l}$ is the turbulent viscosity of liquid phase, Pa·s.

As a dominant interphase force, the drag force is the resistance to the bubbles motion relative to the molten steel and acts opposite to the direction of the bubbles motion. The drag coefficient $C_{D}$ is obtained using a Schiller–Naumann drag equation [22,32].

(12) $$C_{D}=\left\{\begin{array}{cc} \frac{24}{Re_{g}}(1+0.15{Re}_{g}^{0.687}) & \mathrm{if}\;Re_{g}<1000\\ \;0.44 & \mathrm{if}\;Re_{g}\geq 1000 \end{array}\right.$$

with

(13) $$Re_{g}=\rho_{l}\left|{\vec{u}}_{g}-{\vec{u}}_{l}\right|d_{g}/\mu_{l}$$

where $Re_{g}$ is the bubble Reynolds number. $\mu_{l}$ is the viscosity of liquid phase, Pa·s.

The lift force can be described as the function of cross product of the slip velocity and the liquid velocity curl and acts perpendicular to the direction of the relative movement of the bubbles and the liquid. The lift coefficient $C_{L}$ is equal to 0.1 [13,33].

The virtual mass force arises from the inertia of liquid phase relative to the acceleration of bubbles. The virtual mass coefficient $C_{\mathrm{VM}}$ is 0.5 [32,33].

There is the hydrodynamic pressure difference near the wall when gas bubbles flow up in the up-snorkel. The wall lubrication force can push the gas bubbles away from the wall. The wall lubrication force coefficient $C_{\mathrm{WL}}$ is given by the following equation [35]:

(14) $$C_{\mathrm{WL}}=C_{W3}\cdot \max\left\{0,\frac{1-y_{W}/\left(C_{\mathrm{WC}}d_{g}\right)}{C_{\mathrm{WD}}y_{W}{\left[(y_{W}/\left(C_{\mathrm{WC}}d_{g}\right)\right]}^{p^{\prime }-1}}\right\}$$

(15) $${\;C}_{W3}=\left\{\begin{array}{cc} e^{-0.933Eo+0.179} & \mathrm{if}\;1\leq Eo\leq 5\\ 0.001(6Eo-18.7) & \mathrm{if}\;5<Eo\leq 33\\ 0.179 & \mathrm{if}\;33>Eo \end{array}\right.$$

(16) $$Eo=g\left|\rho_{g}-\rho_{l}\right|d_{g}^{2}/\sigma$$

where $y_{W}$ is the wall distance, m, $C_{\mathrm{WC}}$ = 10.0, $C_{\mathrm{WD}}$ = 6.8, and $p^{\prime }$ = 1.7. $C_{W3}$ is related to the Eotvos number Eo, σ is the surface tension coefficient, N/m, and ${\vec{n}}_{W}$ is the outward unit normal vector to the wall.

#### 2.2.3. Tracer Transfer Equation

The mixing time can be determined by solving the tracer transfer equation. The tracer transport can be described by conductive transfer and diffusion transfer.

(17) $$\frac{\partial (\rho_{l}\phi_{T})}{\partial t}+\nabla \cdot (\rho_{l}{\vec{u}}_{l}\phi_{T})=\nabla \cdot (\rho_{l}D_{\mathrm{eff}}\nabla \phi_{T})$$

where $\phi_{T}$ is the tracer concentration, and *t* is the time, s. $D_{\mathrm{eff}}$ is effective diffusion coefficient, m^2^/s.

### 2.3. Numerical Details

**Figure 1:**
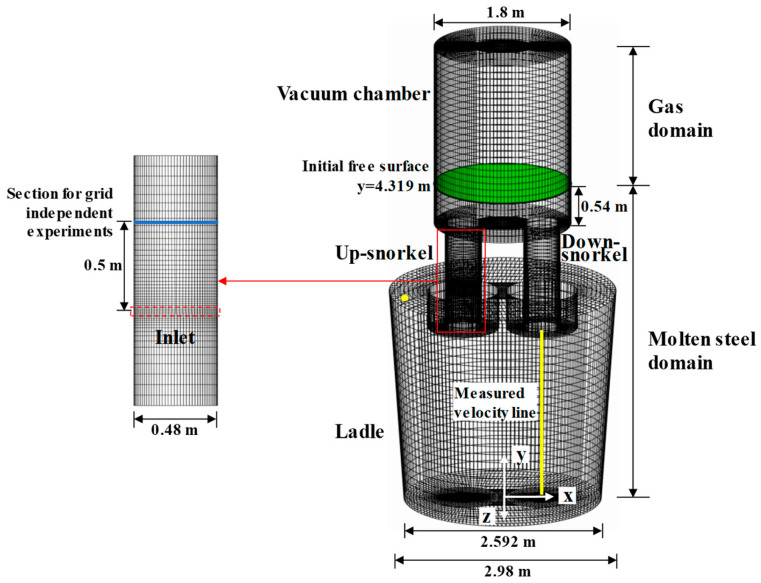
Grid system and inlet location in RH reactor.

There is a traditional RH reactor in Figure 1. The computational domain is covered by non-uniform hexahedral grids. Especially, there is a refined mesh in the up-snorkel because of significant changes in the fluid velocity, and the flow field and the tracer field share the same grid system. The related geometric parameters are listed in Table 1. The governing equations are solved by using the free open source software OpenFOAM-6. The solver for the multiphase flow is the modified reactingTwoPhaseEulerFoam, and the scalarTransportFoam is developed to solve the tracer transfer equation. The convergence criterion is that the root-mean-square-normalized residuals are lower than 10^−5^.

It should be noted that there are two computational domains. One is the molten-steel computational domain [29,30,31,32,33]. The other is the molten-steel–argon-gas computational domain. The molten-steel computational domain only covers the molten-steel domain, while the molten-steel–argon-gas computational domain covers both the molten-steel domain and the argon gas domain.

The interface between the molten steel and the gas in the vacuum chamber is marked by a green color in Figure 1, and the related height is 0.54 m. In the previous paper, the computational domain only consists of the molten-steel domain, and the interface is flat. In the current paper, there are the molten-steel domain and the gas domain, and the interface can move freely in the vacuum chamber.

For multiphase flow, the inlet velocity can be defined by the argon gas flow velocity of every nozzle. The pressure outlets are applied at the outlet of the vacuum chamber. The no-slip condition is applied at the walls, whereas the slip condition is applied at the free surface in the ladle. The tracer transfer is based on the steady fluid flow in RH. The zero gradient is applied at the walls and the outlet, and the fixed values are applied at the inlet. The detailed information can be found in Table 1.

## 3. Model Validation

### 3.1. Validation of Fluid Flow

**Figure 2:**
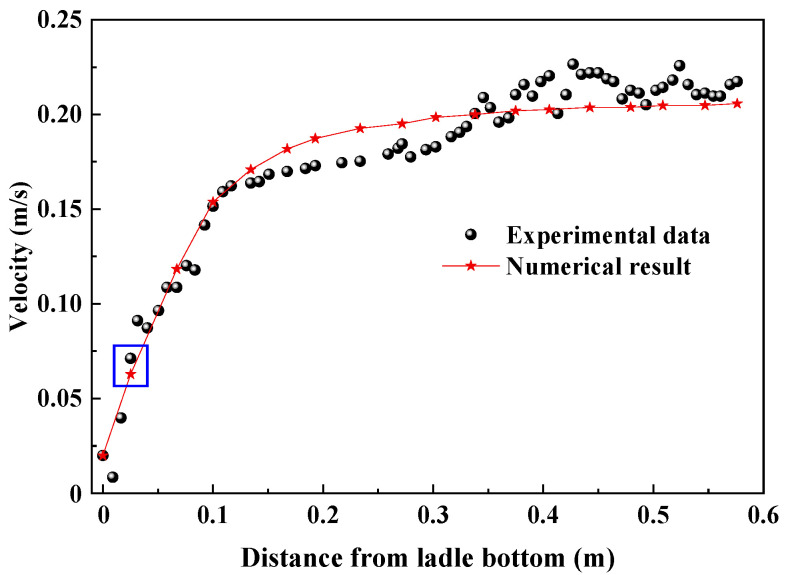
The fluid velocity in the molten-steel–argon-gas computational domain.

Figure 2 gives the simulation results and experimental data of the water model in the case of a gas flow rate of 20 L/min. In Ling’s experiment [13,16,28], PIV (particle image velocimetry) was applied to measure the liquid velocity in the water model with the geometric ratio of 1:5, and the related data were obtained by a 2D image signal acquisition model. The measured velocity line is marked by the yellow color in Figure 1. The simulated velocity distribution agrees well with the measured one. There is the maximum measured velocity 0.226 m/s at a distance of 0.5239 m from ladle bottom, the simulated velocity is 0.205 m/s, and the relative error is 9.3%. Also, there is the maximum relative error 11.75% at the distance of 0.0164 m from the ladle bottom.

### 3.2. Grid-Independent Experiments

In order to make certain that the simulation results are not spurious artifacts of poorly resolved grids, grid-independent experiments are performed in the case of a gas flow rate of 1200 NL/min in the RH reactor. Table 2 lists the maximum velocity of molten steel *v*_max_ and maximum gas volume fraction *α*_gmax_ in the case of five meshes. The horizontal section is located at a distance of 0.5 m above the nozzle in the up-snorkel, which is marked by the blue color in Figure 1. Between the meshes from 172,769 to 210,567, the variations in the maximum velocity of molten steel and the maximum gas volume fraction are 0.025 m/s and 0.026, respectively. In other words, the differences in *v*_max_ and *α*_gmax_ are approximately 1.9% and 12.6%. When the grid is refined from 210,567 to 350,526, the *v*_max_ differences between other adjacent meshes are 0.132 m/s, 0.022 m/s, and 0.008 m/s, and the variations in *α*_gmax_ between the other adjacent meshes are 0.058, 0.008, and 0.005, respectively. Therefore, the 251,214 mesh is sufficient to resolve the Euler–Euler model.

## 4. Results and Discussion

### 4.1. Gas Volume Fraction

**Figure 3:**
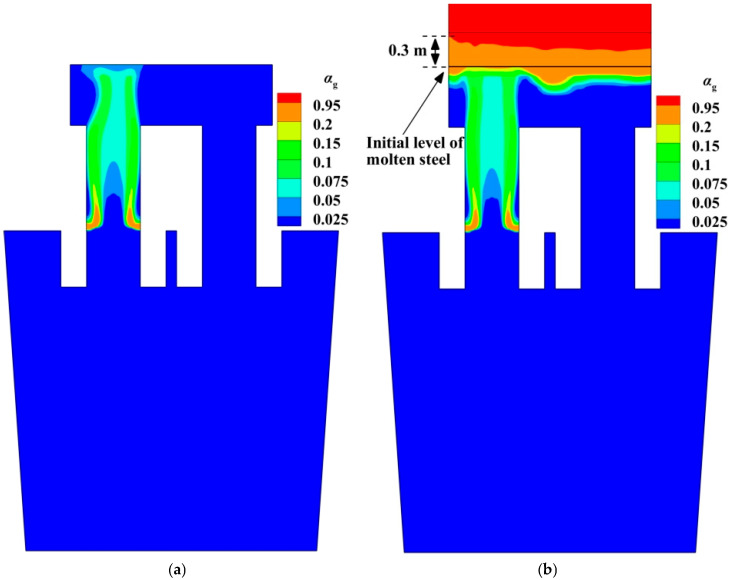
Spatial distribution of gas volume fraction in RH: (**a**) molten steel computational domain; (**b**) molten-steel–argon-gas computational domains.

Figure 3 illustrates the gas volume fraction distribution in RH. The spatial distributions of the gas phase in different computational domains have some similar features: (1) The argon gas is concentrated on the up-snorkel, and there is the higher gas volume fraction near the snorkel wall. (2) After the argon bubbles leave the nozzle, they float vertically upward until they reach the free surface of molten steel in the vacuum chamber. Similar findings are reported in references [9,13,32]. (3) Due to the floatation of the argon bubbles, there are lots of argon bubbles only in the up-snorkel and vacuum chamber. In other words, there is no argon bubble in the down-snorkel and the ladle.

Figure 3 also gives the difference in the gas volume fraction between two computational domains. Figure 3a shows that the bubbles are removed from the molten steel until they touch the interface, but Figure 3b shows that some bubbles can move along the free surface. Figure 3b shows that the interface between the gas and the molten steel is not flat. The bubble column region in the molten steel is the highest, and the region around the bubble column is the lowest. If the gas volume fraction is greater than 0.95, this domain is the gas domain. In this way, the plume domain is about 0.3 m above the initial interface.

### 4.2. Molten-Steel Flow Field

**Figure 4:**
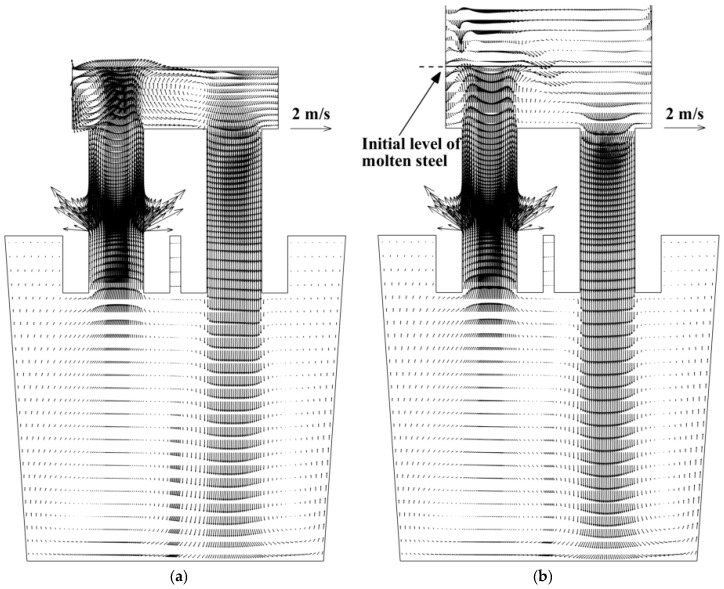
Molten-steel flow field in RH: (**a**) molten steel computational domain; (**b**) molten-steel–argon-gas computational domains.

Figure 4 presents the flow field in RH under the lifting impact of the argon bubbles. The molten steel leaves the up-snorkel as a strong jet flow and flows toward the free surface until it impinges the free surface. Due to the limitation of the free surface, the streams have to flow along the free surface and then form the right recirculation and left recirculation. The strength of the jet flow directly influences the turbulence near the free surface and the degassing effect. Certainly, some of the molten steel flows downwards along the down-snorkel. The molten steel leaves the down-snorkel as a jet flow, spreads outward until it impinges the ladle wall, and then forms two large vortices in the ladle. Because of the limitation of the ladle wall and the snorkel walls, there is a relatively stagnant flow near the free surface in the ladle. The mixing and mass transfer processes in the RH system are predominantly determined by the flow pattern caused by the stirring of the jet flow from the up-snorkel, as well as the large-scale recirculation flow and microscopic vortices.

Figure 4 shows that the flow fields in the ladle, up-snorkel, and down-snorkel are similar, but the flow field in the vacuum chamber is different in the two computational domains. In the molten-steel–argon-gas computational domain, part of the kinetic energy of the jet flow from the up-snorkel can be converted into potential energy, so there are some liquid drops above the free surface, as shown in Figure 3b, and the fluid flow along the free surface becomes weaker. In this way, the center of the vortex between the upward stream and the downward stream is closer to the upward stream, as shown in Figure 4b. In the molten steel computational domain, because of the limitation of the flat free surface in the vacuum chamber, the fluid flow along the free surface is stronger, so the center of the vortex is closer to the upward stream.

### 4.3. Molten-Steel Surface Fluctuation

**Figure 5:**
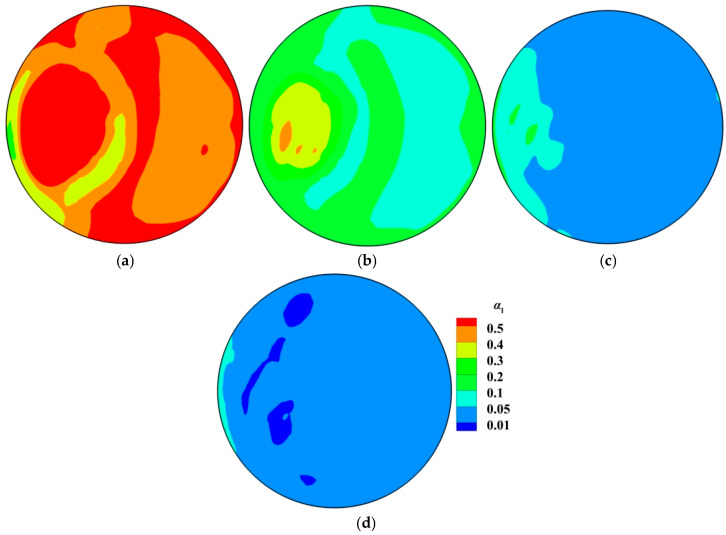
Spatial distribution of molten-steel volume fraction at different cross-sections in vacuum chamber: (**a**) y = 4.319 m; (**b**) y = 4.419 m; (**c**) y = 4.519 m; (**d**) y = 4.619 m.

Figure 5 shows the volume fraction of molten steel in the argon gas domain. The initial free surface is at y = 4.319 m. Figure 5a indicates that the upward flow from the up-snorkel leads to the strong fluctuation of free surface according to the great change in the volume fraction of the liquid phase. Figure 5b shows that the “fountain” formed by the upward flow exceeds 0.1 m above the free surface. Figure 5c,d show that there are still some liquid drops if the distance between the cross-section and the initial free surface is greater than 0.2 m. There are 557 kg droplets above the initial free surface in the vacuum chamber, and then the splashed molten steel is 0.43% of 130 t molten steel in RH.

### 4.4. Mixing Time

**Figure 6:**
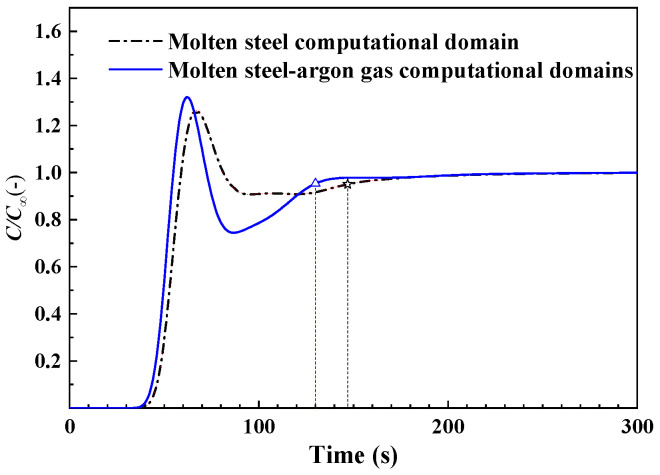
The mixing time described by the tracer mass concentration.

Figure 6 gives the tracer mass concentration at the monitor point, which is marked with a yellow dot in Figure 1. The monitoring point is located 0.16 mm below the free surface of the ladle. The mixing time is regarded as the time that the tracer mass concentration comes to ±5 pct of the finishing concentration (*ϕ_∞_*) [13]. The mixing times are 148 s and 130 s, which are predicted by the molten-steel gas computational domain and the molten-steel–argon-gas computational domain, respectively. There is a difference of up to 12.2% in the mixing time. In other words, the mixing time is underestimated in the molten-steel computational domain.

## 5. Conclusions

In order to describe the motion of the splashed droplets in the RH vacuum chamber, the Euler–Euler model with the inter-phase momentum transfer is applied to investigate the interaction between the molten steel and the bubbles in the RH. The conclusions are as follows:

(1) In previous papers, the computational domain usually only involves the molten-steel domain, so they cannot describe the behavior of the splashed droplets. In the current model, there is a molten-steel domain and a gas domain. In the molten-steel domain, there is an interaction between the molten steel and the argon bubbles. In the gas domain, there is an interaction between the splashed droplets and the gas. Consequently, the computational time rises 20% in two days, but it can give the spatial distribution of the splashed droplets in the vacuum chamber. Such a spatial distribution is very important for the solution of the decarburization model in the future.
(2) There are 557 kg of droplets above the initial free surface of the molten steel in the vacuum chamber in the case of 1200 NL/min, which is up to 0.43% of 130 t of molten steel in the RH, but this model cannot give the size distribution of the splashed droplets.
(3) When the argon domain is considered in the computational domain, the flow fields in the ladle, up-snorkel, and down-snorkel are similar to those in the previous references. Above the free surface of the molten steel in the vacuum chamber, there are ascending and descending droplets.
(4) The “fountain” formed by the upward flow from the up-snorkel exceeds 0.1 m above the free surface of molten steel.
(5) The center of the vortex between the upward stream and the downward stream is closer to the upward stream in vacuum chamber.
(6) The mixing time drops 12.2% after the gas domain is considered in the computational domain.

## Figures and Tables

**Table 1:** Physical parameters.

Parameters	Value
Ladle bottom diameter, m	2.592
Ladle top diameter, m	2.98
Snorkel diameter, m	0.48
Vacuum chamber diameter, m	1.8
Immersion depth, m	0.5
Nozzles diameter, m	0.008
Number of nozzles	4
Inlet flow rate of argon gas, NL/min	1200
Temperature of molten steel, K	1873
Vacuum chamber pressure, Pa	67
Density of molten steel, kg/m^3^	7000
Dynamic viscosity of molten steel, Pa s	6.2 × 10^−3^
Density of argon gas, kg/m^3^	1.783
Dynamic viscosity of argon gas, Pa s	2.39 × 10^−5^
Surface tension between molten steel and argon gas, N/m	1.5
Initial free surface in the vacuum chamber	4.319

**Table 2:** Test of numerical accuracy with different meshes.

Amount of grid cells	172,769	210,567	251,214	305,271	350,526
*α*_gmax_	0.206	0.232	0.29	0.282	0.287
*v*_max_ (m/s)	1.31	1.335	1.467	1.445	1.453

## Data Availability

The original contributions presented in this study are included in the article. Further inquiries can be directed to the corresponding author.
